# Circulating microRNAs, miR-939, miR-595, miR-519d and miR-494, Identify Cirrhotic Patients with HCC

**DOI:** 10.1371/journal.pone.0141448

**Published:** 2015-10-28

**Authors:** Francesca Fornari, Manuela Ferracin, Davide Trerè, Maddalena Milazzo, Sara Marinelli, Marzia Galassi, Laura Venerandi, Daniela Pollutri, Clarissa Patrizi, Alberto Borghi, Francesco G. Foschi, Giuseppe F. Stefanini, Massimo Negrini, Luigi Bolondi, Laura Gramantieri

**Affiliations:** 1 Centro di Ricerca Biomedica Applicata (CRBA), Azienda Ospedaliero-Universitaria Policlinico S. Orsola-Malpighi e Università di Bologna, Bologna, Italy; 2 Dipartimento di Medicina Sperimentale e Diagnostica e Centro Interdipartimentale per la Ricerca sul Cancro, Università di Ferrara, Ferrara, Italy; 3 Dipartimento di Medicina Specialistica, Diagnostica e Sperimentale Università di Bologna, Bologna, Italy; 4 Dipartimento di Medicina Interna, Ospedale per gli Infermi, Faenza, Italy; University of Hong Kong, HONG KONG

## Abstract

The performance of circulating biomarkers for the diagnosis of hepatocellular carcinoma (HCC) is sub-optimal. In this study we tested circulating microRNAs as biomarkers for HCC in cirrhotic patients by performing a two stage study: a discovery phase conducted by microarray and a validation phase performed by qRT-PCR in an independent series of 118 patients. Beside miRNAs emerged from the discovery phase, miR-21, miR-221, miR-519d were also tested in the validation setting on the basis of literary and tissue findings. Deregulated microRNAs were assayed in HCC-derived cells in the intracellular compartment, cell culture supernatant and exosomal fraction. Serum and tissue microRNA levels were compared in 14 patients surgically treated for HCC. From the discovery study, it emerged that seven circulating microRNAs were differentially expressed in cirrhotic patients with and without HCC. In the validation set, miR-939, miR-595 and miR-519d were shown to differentiate cirrhotic patients with and without HCC. MiR-939 and miR-595 are independent factors for HCC. ROC curves of miR-939, miR-595 and miR-519d displayed that AUC was higher than AFP. An exosomal secretion of miR-519d, miR-21, miR-221 and miR-1228 and a correlation between circulating and tissue levels of miR-519d, miR-494 and miR-21 were found in HCC patients. Therefore, we show that circulating microRNAs deserve attention as non-invasive biomarkers in the diagnostic setting of HCC and that exosomal secretion contributes to discharging a subset of microRNAs into the extracellular compartment.

## Introduction

Hepatocellular carcinoma (HCC) is the most frequent primary liver cancer, with an increasing incidence observed over the last decades [1]. Diagnosing HCC in its early stages may strongly affect the therapeutic approach. Imaging techniques such as US, CT and MRI represent the diagnostic approaches recommended by EASL-EORTC and AASLD guidelines. However, in specific cases, nodules smaller than 2 cm in diameter in patients with liver cirrhosis may pose a challenge to non-invasive diagnostics. In cases not characterized by imaging techniques, biopsy is recommended even though it involves the risks of invasive procedures. No circulating biomarker contributing to the early detection or to the staging of HCC is recommended at the moment [2, 3].

Serum AFP was dropped out from guidelines due to its poor sensitivity (39–65%) [4]. Meanwhile, the improvement in the detection capability of imaging techniques, able to identify very small nodular lesions in cirrhotic livers, has made the differential diagnosis of small nodules of uncertain potential an even more relevant issue. Since repeating MRI and CT scans may represent a problem in terms of economic and personnel resources, the availability of reliable biomarkers, to be assayed over time, would represent an aid to the assessment of the malignant potential of liver nodules on cirrhosis. For these reasons, this field of research has been highlighted by both EASL-EORTC and AASLD guidelines as a priority [2, 3].

Circulating microRNAs have been demonstrated to be highly stable in serum and plasma due to their protection from RNase activity, therefore representing a possible source of diagnostic and prognostic biomarkers to be explored. Indeed, miRNAs incorporation in micro-vesicles (e.g. exosomes and apoptotic bodies) or aggregation with RNA-binding proteins (e.g. AGO family members and HDL) protects them from degradation by RNases widely present in body fluids. Several experimental data reported resistance of endogenous circulating miRNAs to severe stressing conditions, such as high temperatures and repeated freeze-thaw cycles, with respect to synthetic miRNAs added to plasma samples which were, by contrast, rapidly degraded [5–8]. In addition, El-Hefnawy et al. [9] showed that plasma RNA is protected from degradation by inclusion in lipid or lipoprotein complexes, but it is destroyed immediately by addition of detergents. This data strongly suggest that extracellular RNA is most likely protected within lipid vesicles, which can be disrupted by detergents.

While consistent experience is available concerning tissue deregulation of miRNAs expression, there is poor consensus regarding a possible diagnostic role of circulating miRNAs in solid tumors and, in particular, in HCC. In addition, few data are available on mechanisms regulating miRNAs release from tumor cells into the bloodstream. In particular, active secretion in protein-bound or membrane-bound complexes [10, 11] or passive release due to tumor lysis have been hypothesized.

Several studies explored the expression of restricted panels of circulating miRNAs in patients with HCC. The majority of these studies were performed by testing serum levels of few miRNAs chosen on the basis of their deregulated expression at the tissue level. However, few evidences sustain any relationship between tissue and circulating miRNA profiles. The studies testing the whole miRNAome to profile circulating miRNAs in HCC patients [12–15] did not obtain homogeneous results. Ultimately, most of the data reported in the literature have been obtained on eastern patients, whose tumor biology might not match that of western patients.

In this study, we investigated the expression of circulating miRNAs in patients with cirrhosis, early and advanced HCC on cirrhosis by using a two-steps approach. A whole microRNAome microarray analysis was applied to explore deregulated miRNAs expression in a discovery set, while RT-qPCR was used to validate the preliminary findings in a prospective and independent cohort of patients. Cirrhotic patients, instead of healthy controls, were chosen as control group because they represent the population enrolled in surveillance programs for early detection of HCC, in which novel biomarkers should be assayed and used. Since the array-based study did not provide any miRNA known to be aberrantly expressed in HCC tissue, we also tested the serum levels of a restricted panel of miRNAs deregulated in HCC lesions [16–18]. Moreover, in order to gain an insight into possible mechanisms involved in the deregulated expression of circulating miRNAs, we analyzed HCC-derived cell lines for the expression of miRNAs in the intracellular compartment, in culture supernatant as well as in its exosomal fraction. Finally, in order to investigate whether circulating miRNAs deregulation mirrored the events occurring at the tissue level, miRNA expression was also analyzed in sera and tumor tissue from 14 patients surgically treated for HCC.

## Patients and Methods

### Study design and patients characteristics

The study was approved by the Institutional Review Board of the S.Orsola-Malpighi University Hospital. A written informed consent was obtained from each patient enrolled in the study. Cirrhotic patients enrolled in this study are prospectively followed at a single center for the diagnosis and treatment of primary liver cancer, either in surveillance programs or in programs aimed at the diagnostic assessment and treatment of nodular liver lesions in cirrhosis. The study was designed as a two phase epidemiological study with a hypothesis generating step conducted by means of microarray assay, aimed at discovering a genome-wide aberrantly regulated circulating miRNA panel able to differentiate cirrhotic (N. 11) from HCC (N. 12) patients. The validation phase was performed on an independent series of 118 consecutive cirrhotic patients: 31 patients without liver nodules, 40 patients with unifocal/small HCC and 47 patients with intermediate (40 cases) or advanced (7 cases) HCC. Patients were prospectively enrolled in the hypothesis generating phase or in the validation phase based on a chronological criteria. Diagnosis and staging of HCC were respectively established according to EASL/EORTC criteria [2] and BCLC classification at the time of diagnosis. The 40 intermediate and the 7 advanced HCCs were grouped together in the statistical analysis, while patients with a single nodule less than 2 cm in diameter were considered as “early” HCCs. The only exceptions were represented by two cases of HCCs that were diagnosed as intermediate HCCs but experienced a neoplastic macrovascular invasion in one case and an extrahepatic spread in the other case, respectively at one month and 2 months follow-up assessment. Thus, in the statistical analysis, they were classified as “advanced” HCCs hypothesizing an under staging at the time of diagnosis. Patients’ characteristics are detailed in Table 1. No patient with cirrhosis developed any nodular lesion within the first 6 months of follow-up. Patients with co-morbidities or complications of the chronic liver disease within the previous 6 months or previous/ongoing anti-cancer or immunomodulatory treatments were excluded from the study. The only exception was represented by anti-viral treatments (nucleotide/nucleosides analogs) in HBV-infected patients. Only Child-Pugh A5 or A6 patients were enrolled, in order to avoid alterations of miRNA profiles associated with liver function impairment. Blood samples were collected and processed according to standard protocols, used for the laboratory assessments in the routine clinical practice. Processing of blood occurred within the first hour after blood drawn.

**Table 1:** Patients characteristics.

		Discovery set (23 pts)	Validation set (115 pts) ^(^ ^1^ ^)^	Difference
**Age**				
	<65 yrs	8 (34.8%)	47 (40.9%)	n.s.
	>65 yrs	15 (65.2%)	68 (59.1%)	n.s.
**Gender**				
	Male	17 (73.9%)	83 (72.2%)	n.s.
	Female	6 (26.1%)	32 (27.8%)	n.s.
**Viral infection**				
	HBV	4 (17.4%)	19 (16.5%)	n.s.
	HCV	17 (73.9%)	81 (70.4%)	n.s.
	None	2 (8.7%)	15 (13.1%)	n.s.
**Stage**				
	cirrhosis	11 (47.8%)	30 (26.1%)	n.s.
	S/U^(^ ^2^ ^)^ HCC	12 (52.2%)	40 (34.8%)	n.s.
	intermediate/advanced HCC		45 (39.1%)	N/A
**Child-Pugh**				
	A5	12 (52.2%)	65 (56.5%)	n.s.
	A6	11 (47.8%)	50 (43.5%)	n.s.
**ALT (NV<41 U/L)**				
	ALT< 41 U/L	1 (4,3%)	8 (7%)	n.s.
	ALT> 41 U/L	22 (95,7%)	107 (93%)	n.s.
**CRP (NV<0.8 mg/dL)** ^(^ ^3^ ^)^				
	CRP<0.8 mg/dL	15 (65.2%)	43 (45.7%)	n.s.
	CRP>0.8 mg/dL	8 (34.8%)	51 (54.3%)	n.s.
**CREA (NV<1.2 mg/dL)**				
	CREA<1.2 mg/dL	20 (86.9%)	91 (79.1%)	n.s.
	CREA>1.2 mg/dL	3 (13.1%)	24 (20.9%)	n.s.
**AFP** ^(^ ^4^ ^)^ **(ng/mL)**				
	< 20 ng/mL	7 (30.4%)	39 (36.1%)	n.s.
	> 20 ng/mL	16 (69.6%)	69 (63.9%)	n.s.

^(1)^ The three patients dropped from the study due to technical reasons were not included in the analysis.

^(2)^ S/U: small/unifocal (less than 2 cm in the main diameter) HCC.

^(3, 4)^ In the validation set, CRP and AFP were not available in 21 and 7 patients, respectively.

Fourteen more patients surgically treated for HCC were tested for miR-939, miR-595, miR-494, miR-1228, miR-519d, miR-21, miR-221 both in serum and HCC tissue. Blood was drawn immediately before surgery. Circulating miRNAs were extracted as described below. Tissue RNA was obtained by using conventional protocols [17].

### Microarray and Real Time PCR analyses

Total RNA was isolated from 400 μl of serum by using Trizol Reagent (Life Technologies) for microarray and RT-qPCR analyses. A synthetic 21-mer RNA (12.5 fmol), which sequence does not match any known human small RNA sequence (cel-miR-39, Ambion), was added after Trizol addition to serum samples and was used for normalization of microarray and qPCR data. Circulating miRNAs were profiled by microarray analysis as described by Lucherini et al. [19]. Briefly, twenty-three samples were hybridized on Agilent human miRNA microarray (#G4470B, Agilent Technologies). This chip consists of 15,000 probes, which represent 723 human microRNAs, sourced from the Sanger miRBase database (Release 10.1). Starting from equal volumes, RNA labeling and hybridization were performed in accordance to manufacturer’s indications. Agilent scanner and the Feature Extraction 10.5 software (Agilent Technologies) were used to obtain the microarray raw-data (data deposited in the public database: Experiment ArrayExpress; Accession code: E-MTAB-3895).

The validation step was performed by RT-qPCR analysis (Taqman microRNA assays, Life Technologies). Total RNA was suspended in 20 μl of DEPC water and 5 μl of 1:3 diluted aliquots were retro-transcribed in cDNA and used for qPCR analysis. Each qPCR reaction was run in triplicate and the average miRNAs expression levels were calculated as previously described for tissue miRNAs [17]. Cel-miR-39 was used as an external control gene and the 2^(-ΔΔCt)^ method was employed to assess miRNA expression. A cel-miR-39 Ct value higher than 30 was considered an exclusion criteria.

In addition to miRNAs emerged from microarray analysis, miR-21, miR-221 and miR-519d were assayed in serum samples of patients enrolled in the validation phase on the basis of previous data reported in the literature.

### Isolation of circulating miRNA from exosomes and cell culture supernatant

MicroRNA levels were measured in the intracellular compartment, in the whole cell culture supernatant as well as in the exosomal fraction of seven HCC-derived cell lines. HepG2 and Hep3B cell lines were cultured with Minimum Essential Medium (Eagle), Huh-7, SNU449, SNU398, SNU182 and SNU475 cells were cultured with RPMI 1640 medium. All media were supplemented with 2 mM Glutamine, 10% FBS and Penicillin-Streptomycin. All the cell lines, except Huh-7 cells, were purchased from ATCC. Huh-7 cells are from Prof. Alberti’s laboratory, University of Padua. For intracellular, extracellular and exosomal miRNAs isolation, cells were grown in full media at a 90% confluence and subsequently in FBS-deprived media for further 48 hours. Total extracellular miRNAs were extracted from 400 μl of cell supernatant. The remaining cell culture supernatant was centrifuged at 500 × g for 10 min at 4°C to remove dead cells and then centrifuged at 20.000 × g to remove large debris. Small debris were eliminated by filtration through 0.2 μm nylon filters. Exosomes pellet was obtained by ultracentrifugation at 100.000 × g for 70 min at 4°C, washed in PBS and collected by ultracentrifugation as above [20]. Exosomes-depleted fraction was represented by 400 μl of PBS washing buffer collected after the last ultracentrifugation. Final pellets containing exosome vesicles were suspended in 400 μl of PBS. Cells were collected from the same flasks used for the detection of extracellular miRNAs. For RNA extraction, Trizol-based protocols specific for cell pellets or serum samples were used. Exosomes isolation from HCC and cirrhotic patients was executed by ultracentrifugation as above described, starting from 400 μl of serum samples.

Western blot analysis of protein extract (10 μg) from cell culture supernatant, exosomes-depleted and enriched fractions was performed by using anti-Alix (Biorad; #MCA2493) and anti-CD63 (Genetex; #GTX37555) specific antibodies. Prime Time qPCR assays were employed to quantify AFP and Albumin mRNAs (IDT; #Hs.PT.56a.571602 and #Hs.PT.56a.1501965, respectively). β-actin was used as housekeeping gene (ACTB_F: 5’-ACCTTCTACAATGAGCTGCG-3’; ACTB_R: 5’-CCTGGATAGCAACGTACATGG-3’).

### Statistical analysis

#### Discovery set

Microarray results were analyzed using the GeneSpring GX 12 software (Agilent Technologies). Data transformation was applied to set all negative raw values at 1.0, followed by normalization on cel-miR-39 levels. A filter on low gene expression was used to keep only the probes expressed in at least one sample. Differentially expressed genes were selected to have a 1.5 fold expression difference between cirrhotic patients with and without HCC and a statistically significant p-value (<0.05), using the Mann-Whitney test. Differentially expressed miRNAs were employed for Cluster Analysis of samples using the Manhattan correlation as a measure of similarity.

#### Validation set

The differential expression of circulating miRNAs emerged from the discovery set was tested by Mann-Whitney U test or Kruskall-Wallis test in subgroups of patients defined on the basis of presence and staging of HCC, viral infection, AFP levels and demographic data. Spearman’s correlation was used to assay any relationship among different miRNAs and between each miRNA and AFP levels. The stepwise backward model selection was performed to assay circulating factors independently associated with the presence of HCC. ROC (Receiver Operating Characteristic) curves were constructed and the AUC (Area Under the Curve) was calculated to explore the diagnostic value of specific miRNAs for differentiating cirrhotic patients with and without HCC.

Pearson’s correlation was used to assay any relationship between intracellular, extracellular and exosomal miRNA levels in HCC-derived cell lines as well as between circulating and intra-tumor miRNA levels in HCC patients. Student’s t-test was employed to analyze any difference in miRNA expression levels between the exosomes-depleted and enriched fraction obtained from patients’ serum samples. All statistical calculations were performed using SPSS 15.0 (SPSS inc).

## Results

### Microarray analysis of the discovery set

**Fig 1:**
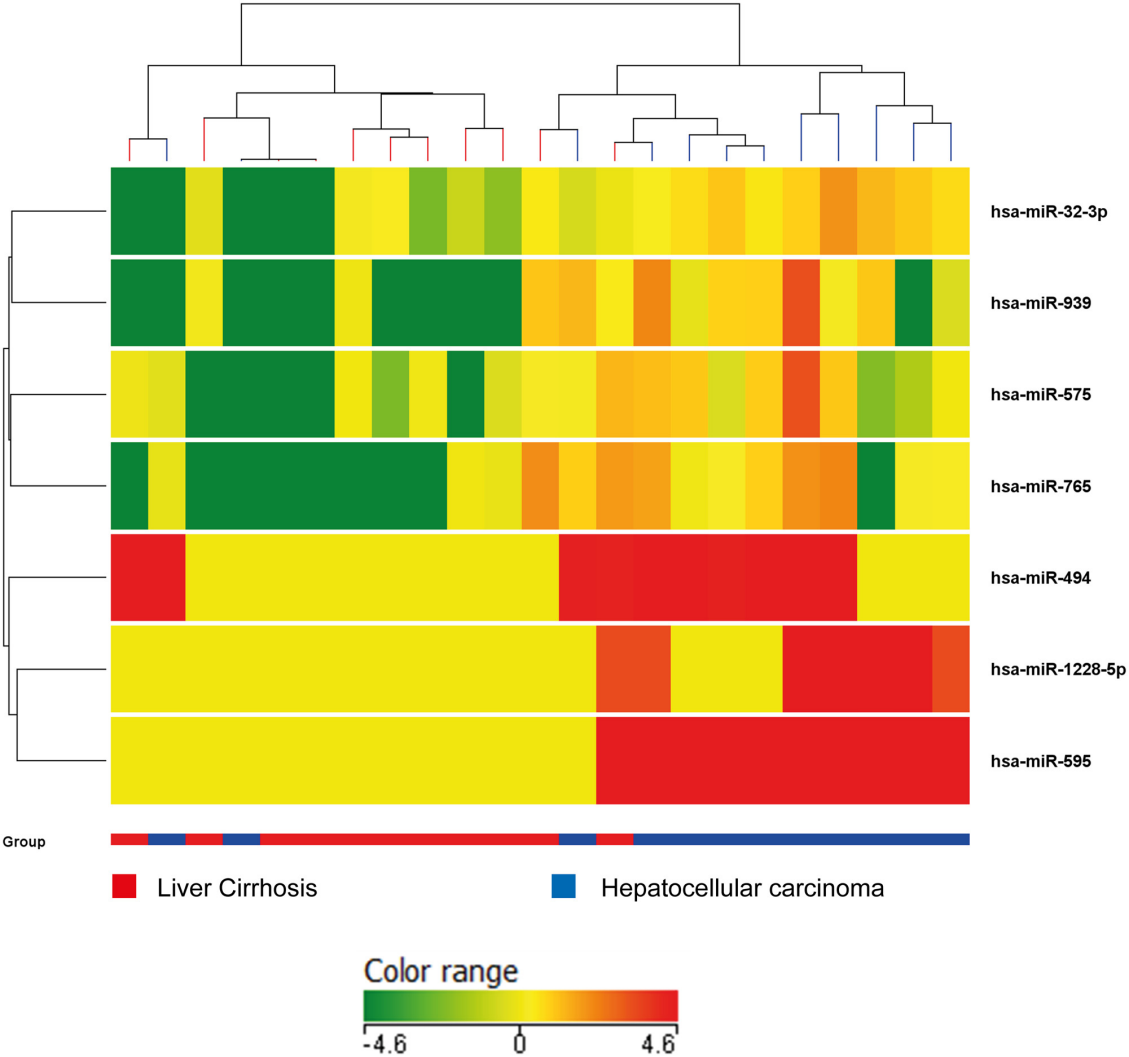
Microarray analysis. Genome-wide circulating miRNA expression analysis in serum of cirrhotic patients with and without HCC. As reported in the expression color-bar, for each miRNA, red color means a higher expression value than its average expression across all samples while green color means a lower expression level than its average expression across all samples.

A total of 122 miRNAs were detected in the serum of 23 patients (11 patients with liver cirrhosis and 12 patients with liver cirrhosis complicated by HCC). Hierarchical cluster analysis showed that the miRNA expression pattern differed between cirrhotic patients with and without HCC. HCC patients displayed an overexpression of seven miRNAs with respect to cirrhotics (p = 0.05 Mann-Whitney test). MiRNAs differentially expressed between cirrhotic patients with and without HCC (hsa-miR-32-3p, hsa-miR-939, hsa-miR-575, hsa-miR-765, hsa-miR-494, hsa-miR-1228-5p, hsa-miR-595) are illustrated in Fig 1. No significantly down-regulated miRNA emerged from the comparison of these two groups.

Data obtained from the array analysis were validated in the same cohort of patients by RT-qPCR. While the reproducibility was very high for miR-595, miR-494, miR-939 and miR-1228 (Spearman’s correlation, p<0.0001 for all of them), serum levels of miR-32-3p turned out to be very low when assessed by RT-qPCR, in the majority of patients, making a reliable quantification questionable. MiR-575 and miR-765 displayed a high variability in this subgroup of cirrhotic and HCC patients, thus they were not tested further. In the light of this preliminary analysis, we proceeded with the assessment of miR-595, miR-494, miR-1228 and miR-939 levels in the validation set.

### Validation study

MiR-595, miR-494, miR-1228 and miR-939 were validated in an expanded case-control study performed on 118 patients with liver cirrhosis (N = 31), unifocal (less than 2 cm in the main diameter) HCCs (N = 40), and intermediate-advanced HCCs (N = 47). In three patients (one patient with cirrhosis and two with advanced HCC) a Ct value higher than 30 was obtained at RT-qPCR for cel-miR-39. Therefore these three cases were dropped out from the study since miRNAs quantification was considered not reliable. Three additional circulating miRNAs chosen on the basis of tissue and literary findings, miR-21, miR-221 and miR-519d, were tested in the validation set even though they did not emerge from the array analysis.

A high correlation was found between circulating miR-939, miR-595 and miR-519d (Spearman’s correlation, p<0.0001), as well as between miR-221 and miR-21 (Spearman’s correlation, p<0.0001), miR-939 and miR-221 (Spearman’s correlation, p<0.0001) and between miR-494 and miR-1228 (Spearman’s correlation, p<0.0001). Similarly, miR-595 displayed a high correlation with miR-221 (Spearman’s correlation, p = 0.002) and miR-21 (Spearman’s correlation, p = 0.006).

**Fig 2:**
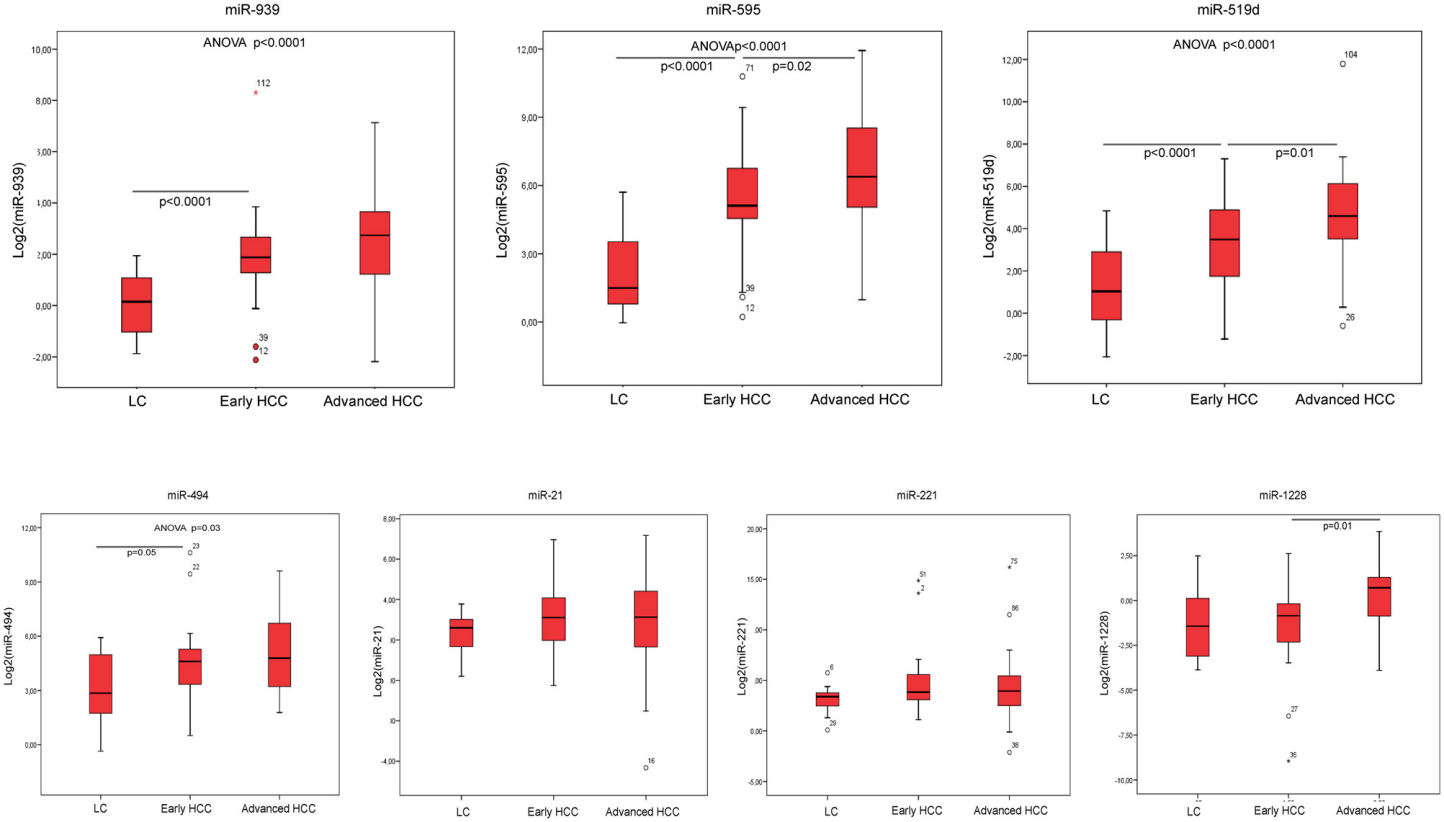
Real Time PCR validation analysis. Box-plot representation of circulating levels of selected miRNAs (miR-939, miR-595, miR-519d, miR-494, miR-21, miR-221) in patients with liver cirrhosis (LC) without nodular liver lesions, or with early HCCs (unifocal HCCs less than 2 cm in the main diameter) or intermediate/advanced HCCs. Graphic representation and statistical analysis were performed after Log2 transformation of RT-qPCR data.

Cirrhotic patients without HCC, with small/unifocal HCC and with intermediate/advanced HCC were compared. Circulating levels of miR-939, miR-595, miR-519d and miR-494 turned out to differentiate these three groups (ANOVA, p<0.0001 for miR-939, miR-595, miR-519d; p = 0.032 for miR-494) while no significant difference was observed for miR-21, miR-221 and miR-1228 according to the presence or absence of HCC. When the levels of circulating miRNAs were compared in cirrhotic patients with or without small/unifocal HCC, again the difference was significant for miR-939, miR-595, miR-519d (Mann-Whitney U test, p<0.0001) and for miR-494 (p = 0.05). Conversely, when small/unifocal HCCs were compared with advanced HCC, only miR-595 (Mann-Whitney U test, p = 0.02), miR-519d (Mann-Whitney U test, p = 0.01) and miR-1228 (Mann-Whitney U test, p = 0.01) differentiated the two groups. These findings are summarized in Fig 2.

Using the stepwise selection model to gradually eliminate non-significant covariates, the logistic regression showed AFP (p = 0.008) and miR-595 (p = 0.01) as the best predictive circulating factors when all patients with HCC were included in the analysis. Higher miR-595 serum levels turned out to be an independent factor for the presence of HCC in advanced stage, or in HCCs with macro-vascular invasion (p = 0.007) with respect to small/unifocal HCCs.

Conversely, when only small/unifocal HCCs entered the analysis, miR-939 was significantly associated with increased HCC risk, compared to cirrhotic patients without nodular liver lesions (p = 0.009). No association was found between higher circulating miRNA levels and the presence of HBV or HCV infection or the presence of other risk factors.

**Fig 3:**
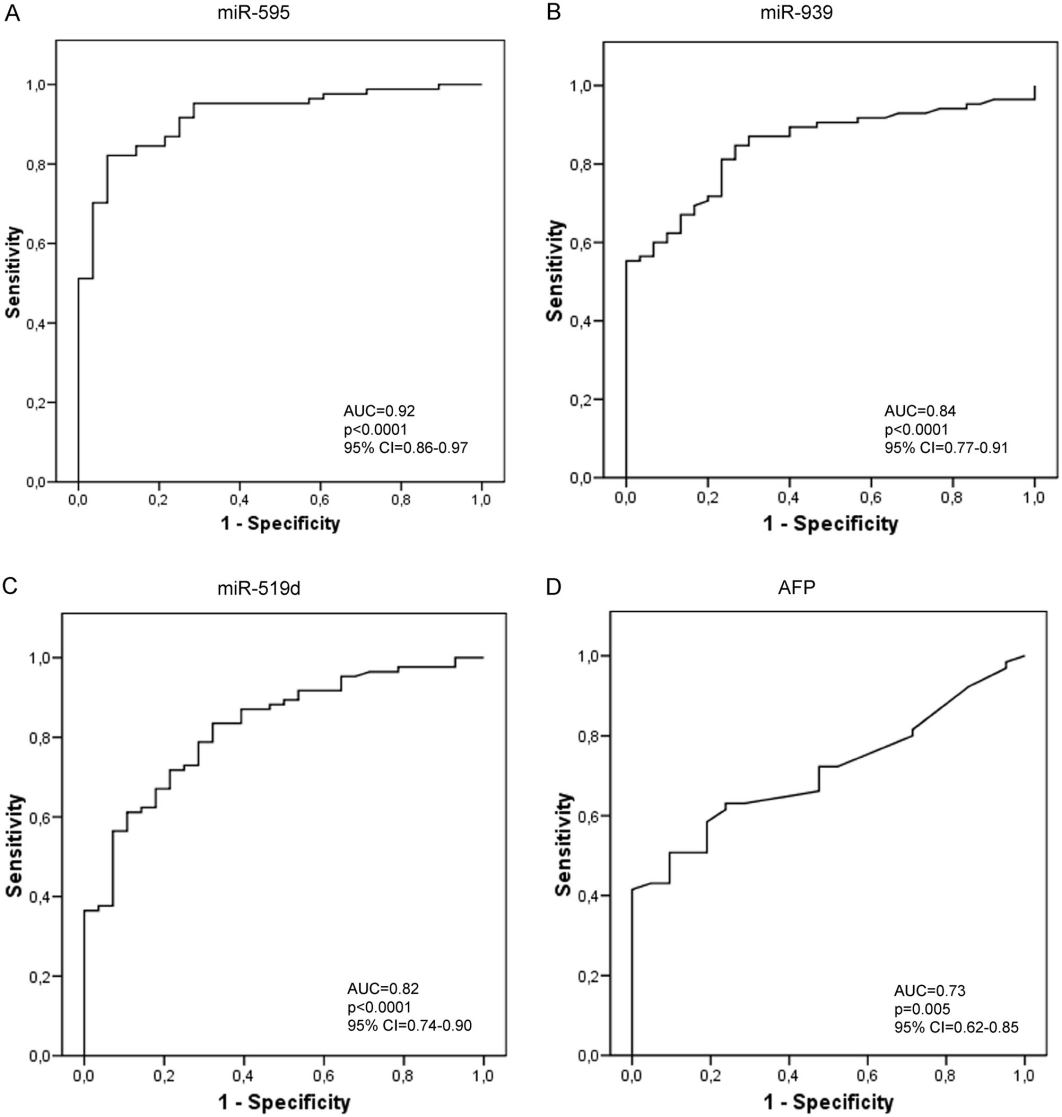
MiRNAs performance as biomarkers of diagnosis. Receiver operating characteristic curve (ROC) analysis for hepatocellular carcinoma diagnosis. Area under the curve (AUC) estimation for miR-595 (**A**), miR-939 (**B**), miR-519d (**C**) and alpha-feto-protein (**D**).

ROC (Receiver Operating Characteristic) curves were constructed and the AUC (Area Under the Curve) was calculated for each miRNA emerged as differentially expressed in patients with HCC and for AFP serum levels. Among the tested miRNAs, miR-595 displayed an AUC of 0.92 (CI: 0.86–0.97), miR-939 displayed an AUC of 0.84 (CI: 0.77–0.91), miR-519d showed an AUC of 0.82 (CI: 0.74–0.90). In the diagnostic setting of HCC on cirrhosis, all these miRNAs appeared to perform better than AFP, which, in our series, displayed an AUC of 0.73 (CI: 0.62–0.85) (Fig 3). Conversely, miR-494 (AUC: 0.55, CI: 0.34–0.74), miR-21 (AUC: 0.64, CI: 0.52–0.76), miR-221 (AUC: 0.64, CI: 0.51–0.76) and miR-1228 (AUC: 0.62, CI: 0.45–0.79) displayed a worse performance when compared to AFP.

### MiRNAs secretion mechanisms in HCC-derived cell lines

**Fig 4:**
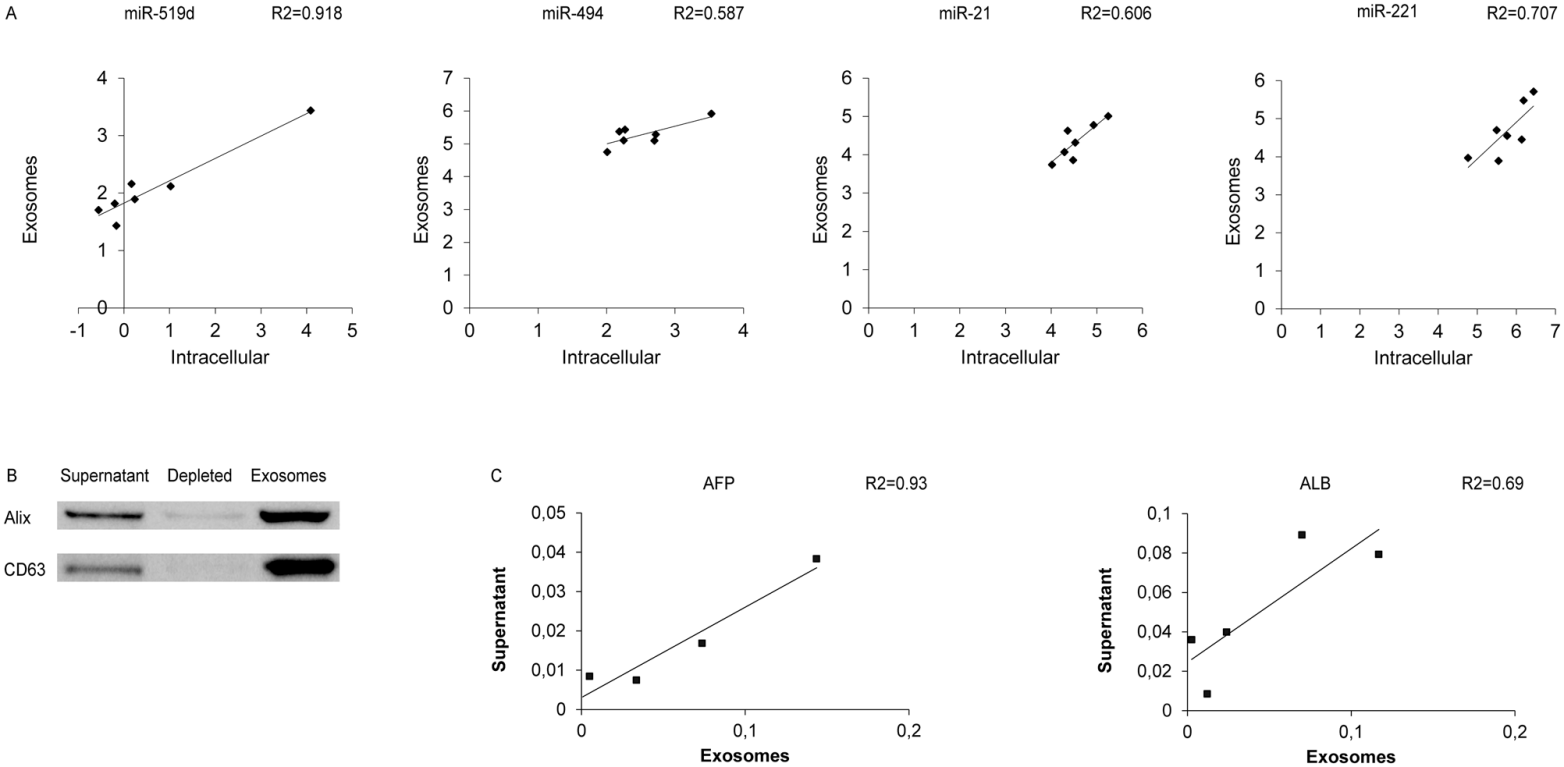
Exosome characterization in HCC cell lines. (A) Correlation graphs between intracellular levels and exosomal fraction of miR-519d, miR-494, miR-21, miR-221 in 7 HCC-derived cell lines. Numbers in x- and y-axes represent 2^-ΔΔCt^ values from the Real Time PCR analysis, transformed in a logarithmic form. U6RNA was used as housekeeping gene for the quantification of intracellular miRNAs levels, whereas cel-miR-39 was used to normalize data derived from exosome miRNA analysis. (B) Western blot analysis of exosomes-specific markers, Alix and CD63, in cell culture supernatant, exosomes-depleted fraction and exosomal fraction derived from Huh-7 cell line. (C) Correlation graphs between cell culture supernatant and exosomal fraction of AFP and Albumin (ALB) mRNA levels in HCC-derived cell lines. β-actin was used as housekeeping gene. Numbers in the y-axis represent 2^-ΔΔCt^ values from the Real Time PCR analysis reported in a linear scale.

To get an insight into possible mechanisms sustaining aberrant circulating miRNA levels, the intracellular expression of these miRNAs was compared with their extracellular expression in cell culture supernatant and in the exosomal fraction in HCC-derived cell lines. A correlation was found between intracellular and exosomal levels of miR-519d (R2 = 0.92; Pearson’s correlation p = 0.001), miR-494 (R2 = 0.59; Pearson’s correlation p = 0.044), miR-221 (R2 = 0.71; Pearson’s correlation p = 0.018) and miR-21 (R2 = 0.61; Pearson’s correlation p = 0.039), suggesting exosomal secretion as a mechanism that contributes to their increased circulating levels (Fig 4A). Notably, a significant correlation between miRNA levels in cell culture supernatant and its enriched exosomal fraction was obtained for miR-519d (R2 = 0.84; Pearson’s correlation p = 0.003), miR-1228 (R2 = 0.78; Pearson’s correlation p = 0.024) and miR-21 (R2 = 0.42; Pearson’s correlation p = 0.05), which allowed us to hypothesize an active vesicle-mediated way of secretion for these miRNAs.

In order to verify the performance of the exosome extraction protocol, Western blot analysis of exosome-specific markers was performed in HCC-derived cell lines. In particular, CD63 and Alix proteins were tested in the cell culture supernatant, in its exosomal fraction as well as in the supernatant depleted from exosomes. As shown in Fig 4B, these two exosomal markers were found in high concentration in the exosomal fraction whereas they were barely detectable in the cell culture supernatant depleted from exosomes. In addition, to investigate whether exosomes might contain HCC-specific mRNAs, Albumin and AFP mRNAs were analyzed by qPCR in cell culture supernatant, as well as in the exosomes-depleted and enriched fractions from HCC-derived cell lines. MRNA expression of these two genes was detected in both cell culture supernatant and exosomal fraction but not in the exosome-depleted fraction. As shown in Fig 4C, a positive correlation between cell culture supernatant and exosomes mRNA levels was found for AFP but not for Albumin mRNA (Pearson’s correlation p = 0.036 and p = 0.083, respectively).

### Relationship between circulating and intracellular miRNAs in HCC patients and their secretion in the exosomal compartment

**Fig 5:**
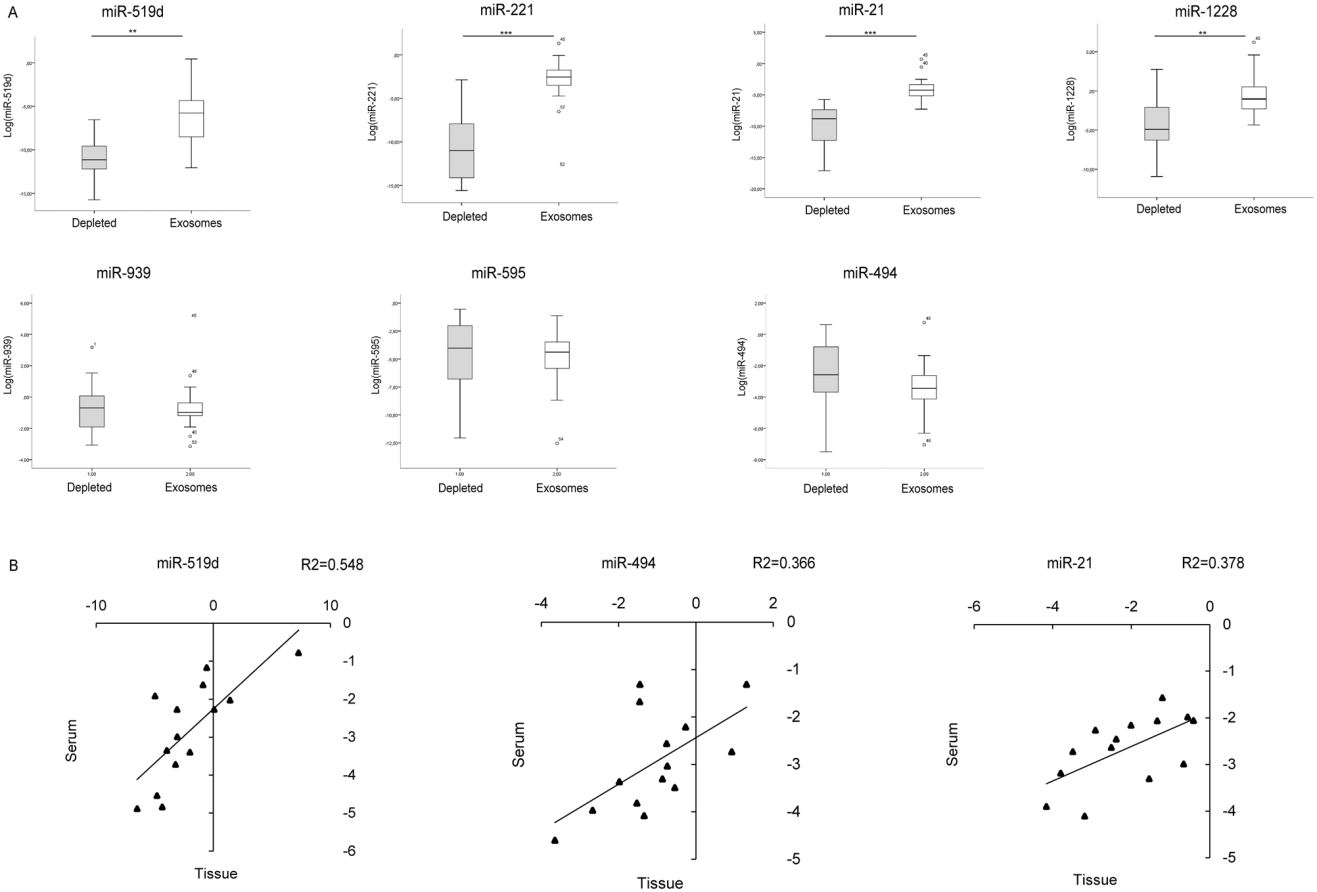
Exosomal and tissue miRNAs quantification in HCC patients. (**A**) Box plot graphs showing miRNA levels in the exosomal-depleted and enriched fractions. Numbers in the y-axis represent 2^-ΔΔCt^ values from the Real Time PCR analysis, transformed in a logarithmic form. ** p<0.01; *** p<0.0001. (**B**) Correlation graphs between intra-tumor and serum levels of miR-519d, miR-494 and miR-21 in 14 HCC patients. Graphic representation and statistical analysis were performed after Log2 transformation of RT-qPCR data. Numbers in x- and y-axes represent 2^-ΔΔCt^ values from the Real Time PCR analysis, transformed in a logarithmic form. U6RNA was used as housekeeping gene for the quantification of tissue miRNAs levels, whereas cel-miR-39 was used to normalize data derived from serum miRNA analysis.

In order to investigate whether the in vitro findings matched the events occurring in HCC patients, the same miRNAs were quantified in the exosomal and exosomes-depleted serum fractions from 30 patients (10 patients with liver cirrhosis without nodular liver lesions, 13 patients with early HCC and 7 patients with advanced HCC). High expression levels of miR-519d, miR-21, miR-221 and miR-1228 were detected in the exosomal fraction, whereas nearly absent levels were observed in the exosomes-depleted fraction (Student’s t-test p = 0.001, p<0.0001, p<0.0001, p = 0.002 respectively). No difference in terms of miR-595, miR-939 and miR-494 levels was found between the exosomal and exosomes-depleted fraction (Fig 5A). These preliminary data allowed us to hypothesize different mechanisms of release of circulating miRNAs, which appeared to be exosome-mediated for miR-519d, miR-21, miR-221 and miR-1228.

Subsequently, to shed light on possible relationships between tumor and circulating miRNA levels, fourteen patients subjected to surgical resection for HCC on cirrhosis were tested for miRNAs expression in serum and HCC tissue. A correlation was found between tissue and serum levels of miR-519d (R2 = 0.55; Pearson’s correlation, p = 0.003), miR-494 (R2 = 0.37; Pearson’s correlation, p = 0.022) and miR-21 (R2 = 0.38; Pearson’s correlation, p = 0.019), while no correlation was found for miR-939, miR-595, miR-221 and miR-1228 (Fig 5B).

## Discussion

Non invasive biomarkers helpful in the diagnostic and prognostic assessment of HCC are lacking and the research in this field is strongly encouraged [2, 3].

Circulating miRNAs profiling in serum or plasma relies upon their stability and reproducibility of their quantification. Specific circulating miRNAs profiles were described in several diseases such as diabetes, lung, ovarian and colorectal cancers [5]. HCC diagnostic and prognostic assessment represents a particularly suitable setting to test circulating miRNAs performance due to: (1) the presence of a high risk population under surveillance, (2) the lack of reliable circulating biomarkers, (3) the possible difficulties in obtaining bioptic samples in cirrhotic patients with liver nodules of uncertain malignant potential identified with imaging techniques, (4) the prognostic impact of early diagnosis of HCC.

Several studies have been published on this topic in the recent years, however there is poor consensus regarding circulating miRNAs profiles in patients with HCC. This could be ascribed to heterogeneous study designs (one-step or two- or more steps studies), analytical approaches (array or RT-qPCR-based assays), choice of internal or exogenous control genes/miRNAs, body fluid to test (serum or plasma), choice of control populations, sample size, normalization protocols, different etiologies and prevalence of risk factors. Remarkably, most of the studies published so far have been mainly run on HBV-related liver diseases while our patients population is mostly HCV-infected. We could not identify any significant correlation between etiology (hepatitis viral infections, presence of metabolic syndrome features, absence of viral infections, etc…) and different miRNAs patterns. This might be ascribed to the small number of patients available in the different subgroups.

In our study, the up-regulation of a panel of circulating miRNAs was the most relevant event associated with the presence of HCC in the exploratory setting. Similar findings were reported by Shen et al [21]. Conversely, other studies [12] found also a down-regulation of specific miRNAs. This might be ascribed to the choice of control groups (represented by cirrhotics in our study and by healthy controls or patients with chronic hepatitis in most of previously reported series) as well as to the different prevalence of risk factors, such as HBV and HCV infection. Indeed, microRNAs can be released into the bloodstream not only by tumor cells, but also by cells of the immune system and those of the peri-tumoral micro-environment, which are differentially activated in relation with etiologies and stages of the chronic liver disease.

The present study was designed as a two-phases study with a discovery step, based on microarray analysis, followed by a RT-qPCR-based validation step in an enlarged, independent series of patients. The control population is represented by cirrhotic patients without nodular liver lesions. Indeed, since these patients are followed in surveillance programs they might take advantage from the discovery of novel non-invasive biomarkers for HCC. The body fluid analyzed here is the serum because it is routinely assayed in the clinical work-up of patients. These experimental choices were taken in the perspective of a possible translation of findings in the clinical practice.

Our whole-genome miRNA analysis in sera from cirrhotic patients with and without HCC identified a panel of 7 miRNAs overexpressed in HCC. The further validation of four of them confirmed that miR-939, miR-595 and miR-494 differentiate cirrhotic patients with and without HCC, while no significant difference was observed for miR-1228. Three more miRNAs, namely miR-21, miR-221 and miR-519d, were assayed on the basis of tissue findings as well as on the basis of previous reports. MiR-519d elevation in the serum of patients with HCC is in line with tissue data previously reported by our group [16]. Remarkably, miR-519d turned out to differentiate cirrhotic patients without HCC from cirrhotic patients with early and intermediate-advanced HCC. Moreover, miR-519d overexpression differentiates cirrhotic patients with or without small/unifocal HCC as well as cirrhotic patients with small/unifocal and intermediate/advanced HCCs.

On the contrary, findings on circulating miR-21 and miR-221 do not match their overexpression in HCC nodules as reported in previous studies, which were however run in different populations in terms of viral prevalence, ethnicity and by means of different study approaches. Indeed findings on circulating miR-21 in patients with HCC are heterogeneous. Tomimaru and colleagues [22] found increased miR-21 circulating levels in patients with HCC, regardless of the presence of cirrhosis or viral status. In that study, however, HCC patients were compared with healthy volunteers or patients with chronic hepatitis, while a comparison between cirrhotic patients with or without HCC was not performed. These aspects, together with the use of plasma instead of serum and the choice of miR-16 as an internal miRNA control instead of the exogenous cel-miR-39, might account for the apparent discrepancy with our findings. Conversely, Xu et al. [23] reported lower serum levels of miR-21 in patients with HCC when compared with patients with chronic hepatitis. Similarly, Qi et al. [13] confirmed an absence of miR-21 increase in HBV-related HCC patients. Thus, circulating miR-21 levels do not allow the differentiation between patients with cirrhosis and patients with cirrhosis complicated by HCC.

MiR-221 serum levels were reported to be increased in patients with HCC [24]. Even though a first look excludes a possible match with our findings, we should consider that the study by Li and coworkers compared HCC patients with healthy subjects and, remarkably, a strong association between high miR-221 levels and presence of cirrhosis was outlined in that study. The high miR-221 circulating levels in cirrhotic patients may justify the discrepancy between that study and the present one, which included cirrhotic patients as a control group.

Among the studies performed by using a two- or more steps approach, Gui et al [14] identified a panel of miRNAs up-regulated in patients with liver cirrhosis or HCC. The choice of healthy controls which were compared with both HCC and cirrhotic patients as well as the high prevalence of HBV infection, might account for the discrepancy with our results. The same considerations apply to the study of Shen and coworkers [21] that however identified increased circulating levels of miR-520b, belonging to the Chromosome 19 MiRNA Cluster (C19MC) which also includes miR-519d. At the tissue level, several miRNAs of this cluster are up-regulated in HCC, thus it is conceivable that the same might apply for the circulating fraction of C19MC miRNAs. A very relevant study on circulating miRNAs conducted by Zhou et al. [12] in a wide cohort of Asiatic patients infected by HBV at different stages of the liver disease, identified a panel of deregulated circulating miRNAs displaying a high diagnostic accuracy for HCC. Interestingly, despite this study was performed on a different study population, yet high circulating miR-595 and miR-765 levels were identified among miRNAs differentiating cirrhotic patients with and without HCC. The same two miRNAs were found in the seven-miRNAs panel identified by cluster analysis and differentiating cirrhotic patients with and without HCC in our series. These findings are very interesting since they were obtained in a study population not matching with ours, at least as far as etiology and the genetic background are concerned. Additionally, in the above mentioned study, plasma, instead of serum, was analyzed. Even though in our series miR-765 displayed a high variability in a preliminary assay run on a subgroup of cirrhotic patients with and without HCC, circulating miR-595 and miR-765 hold promise as HCC-related circulating miRNAs discriminating cirrhotic patients with and without HCC.

Circulating levels of three miRNAs overexpressed in sera from HCC patients, miR-519d, miR-494 and miR-21, correlated with their HCC tissue levels. Furthermore, our data supported an exosomal secretion for miR-519d, miR-21, miR-221 and miR-1228. Exosomal secretion is considered a relevant event in tumor biology, due to its crucial role in intercellular communications, either supporting or perturbing pathophysiological processes. Indeed, exosomes secreted by tumor cells were demonstrated to deliver oncogenes, pathogens and microRNAs which, in this compartment, are more stable [25]. Abnormal levels of circulating miRNAs in patients with HCC on liver cirrhosis might be due to several factors besides lysis and active secretion by cancer cells. For instance, tumor surrounding or infiltrating cells and the “field effect” might participate to increasing the levels of circulating miRNAs, too. Notably, the contribution of tumor surrounding tissue and infiltrating cells, beside that of tumor cells, should be considered as a relevant source of abnormal levels of specific circulating miRNAs as suggested by recent findings reported on miR-494 in tumor-expanded myeloid suppressor cells [26]. In addition, recent findings by Fabbri and coworkers [27] demonstrated a hormone-like action for circulating miR-21 and miR-29, activating toll-like receptors-mediated pro-inflammatory response. Therefore, additional studies are necessary to explore mechanisms and cell types contributing to circulating miRNAs levels.

In conclusion, even if further validation studies are needed, here we demonstrate that circulating microRNAs deserve attention as potential non-invasive biomarkers for HCC in the diagnostic setting.

## Abbreviations

HCC: hepatocellular carcinoma
miRNA: microRNA
AFP: alpha-feto-protein
qRT-PCR: quantitative Retro-Transcription Polymerase Chain Reaction
ROC: Receiver Operating Characteristic
AUC: Area Under the Curve
US: Ultrasound
CT: Computed Tomography
MRI: Magnetic Resonance Imaging
EASL-EORTC: European Association for the Study of the Liver- European Organization for Research and Treatment of Cancer
AASLD: American Association for the Study of Liver Diseases
HBV: Hepatitis B Virus
HCV: Hepatitis C Virus
Ct: Cycle threshold
C19MC: Chromosome 19 MiRNA Cluster

## References

[1] Forner A, Llovet JM, Bruix J. Hepatocellular carcinoma. Lancet. 2012;379(9822):1245–55. 10.1016/S0140-6736(11)61347-0 22353262 PMC13537732

[2] EASL-EORTC clinical practice guidelines: management of hepatocellular carcinoma. J Hepatol. 2012;56(4):908–43. 10.1016/j.jhep.2011.12.001 22424438

[3] Bruix J, Sherman M. Management of hepatocellular carcinoma: an update. Hepatology. 2010;53(3):1020–2. 10.1002/hep.24199 PMC3084991 21374666

[4] Collier J, Sherman M. Screening for hepatocellular carcinoma. Hepatology. 1998;27(1):273–8. 9425947 10.1002/hep.510270140

[5] Chen X, Ba Y, Ma L, Cai X, Yin Y, Wang K, et al Characterization of microRNAs in serum: a novel class of biomarkers for diagnosis of cancer and other diseases. Cell Res. 2008;18(10):997–1006. 10.1038/cr.2008.282 18766170

[6] Mitchell PS, Parkin RK, Kroh EM, Fritz BR, Wyman SK, Pogosova-Agadjanyan EL, et al Circulating microRNAs as stable blood-based markers for cancer detection. Proc Natl Acad Sci U S A. 2008;105(30):10513–8. 10.1073/pnas.0804549105 18663219 PMC2492472

[7] Schwarzenbach H, Nishida N, Calin GA, Pantel K. Clinical relevance of circulating cell-free microRNAs in cancer. Nature reviews Clinical oncology. 2014;11(3):145–56. 10.1038/nrclinonc.2014.5 24492836

[8] Kosaka N, Iguchi H, Ochiya T. Circulating microRNA in body fluid: a new potential biomarker for cancer diagnosis and prognosis. Cancer science. 2010;101(10):2087–92. 10.1111/j.1349-7006.2010.01650.x 20624164 PMC11159200

[9] El-Hefnawy T, Raja S, Kelly L, Bigbee WL, Kirkwood JM, Luketich JD, et al Characterization of amplifiable, circulating RNA in plasma and its potential as a tool for cancer diagnostics. Clinical chemistry. 2004;50(3):564–73. 14718398 10.1373/clinchem.2003.028506

[10] Arroyo JD, Chevillet JR, Kroh EM, Ruf IK, Pritchard CC, Gibson DF, et al Argonaute2 complexes carry a population of circulating microRNAs independent of vesicles in human plasma. Proc Natl Acad Sci U S A. 2011;108(12):5003–8. 10.1073/pnas.1019055108 21383194 PMC3064324

[11] Nilsson RJ, Balaj L, Hulleman E, van Rijn S, Pegtel DM, Walraven M, et al Blood platelets contain tumor-derived RNA biomarkers. Blood. 2011;118(13):3680–3. 10.1182/blood-2011-03-344408 21832279 PMC7224637

[12] Zhou J, Yu L, Gao X, Hu J, Wang J, Dai Z, et al Plasma microRNA panel to diagnose hepatitis B virus-related hepatocellular carcinoma. J Clin Oncol. 2011;29(36):4781–8. 10.1200/JCO.2011.38.2697 22105822

[13] Qi P, Cheng SQ, Wang H, Li N, Chen YF, Gao CF. Serum microRNAs as biomarkers for hepatocellular carcinoma in Chinese patients with chronic hepatitis B virus infection. PLoS One. 2011;6(12):e28486 10.1371/journal.pone.0028486 22174818 PMC3234251

[14] Gui J, Tian Y, Wen X, Zhang W, Zhang P, Gao J, et al Serum microRNA characterization identifies miR-885-5p as a potential marker for detecting liver pathologies. Clin Sci (Lond). 2011;120(5):183–93. 20815808 10.1042/CS20100297 PMC2990200

[15] Yamamoto Y, Kosaka N, Tanaka M, Koizumi F, Kanai Y, Mizutani T, et al MicroRNA-500 as a potential diagnostic marker for hepatocellular carcinoma. Biomarkers. 2009;14(7):529–38. 10.3109/13547500903150771 19863192

[16] Fornari F, Milazzo M, Chieco P, Negrini M, Marasco E, Capranico G, et al In hepatocellular carcinoma miR-519d is up-regulated by p53 and DNA hypomethylation and targets CDKN1A/p21, PTEN, AKT3 and TIMP2. J Pathol. 2012;227(3):275–85. 10.1002/path.3995 22262409

[17] Gramantieri L, Ferracin M, Fornari F, Veronese A, Sabbioni S, Liu CG, et al Cyclin G1 is a target of miR-122a, a microRNA frequently down-regulated in human hepatocellular carcinoma. Cancer Res. 2007;67(13):6092–9. 17616664 10.1158/0008-5472.CAN-06-4607

[18] Meng F, Henson R, Wehbe-Janek H, Ghoshal K, Jacob ST, Patel T. MicroRNA-21 regulates expression of the PTEN tumor suppressor gene in human hepatocellular cancer. Gastroenterology. 2007;133(2):647–58. 17681183 10.1053/j.gastro.2007.05.022 PMC4285346

[19] Lucherini OM, Obici L, Ferracin M, Fulci V, McDermott MF, Merlini G, et al First report of circulating microRNAs in tumour necrosis factor receptor-associated periodic syndrome (TRAPS). PLoS One. 2013;8(9):e73443 10.1371/journal.pone.0073443 24066048 PMC3774691

[20] Peinado H, Aleckovic M, Lavotshkin S, Matei I, Costa-Silva B, Moreno-Bueno G, et al Melanoma exosomes educate bone marrow progenitor cells toward a pro-metastatic phenotype through MET. Nat Med. 2012;18(6):883–91. 10.1038/nm.2753 22635005 PMC3645291

[21] Shen J, Wang A, Wang Q, Gurvich I, Siegel AB, Remotti H, et al Exploration of genome-wide circulating microRNA in hepatocellular carcinoma: MiR-483-5p as a potential biomarker. Cancer Epidemiol Biomarkers Prev. 2013;22(12):2364–73. 10.1158/1055-9965.EPI-13-0237 24127413 PMC3963823

[22] Tomimaru Y, Eguchi H, Nagano H, Wada H, Kobayashi S, Marubashi S, et al Circulating microRNA-21 as a novel biomarker for hepatocellular carcinoma. J Hepatol. 2012;56(1):167–75. 10.1016/j.jhep.2011.04.026 21749846

[23] Xu J, Wu C, Che X, Wang L, Yu D, Zhang T, et al Circulating microRNAs, miR-21, miR-122, and miR-223, in patients with hepatocellular carcinoma or chronic hepatitis. Mol Carcinog. 2011;50(2):136–42. 10.1002/mc.20712 21229610

[24] Li J, Wang Y, Yu W, Chen J, Luo J. Expression of serum miR-221 in human hepatocellular carcinoma and its prognostic significance. Biochem Biophys Res Commun. 2011;406(1):70–3. 10.1016/j.bbrc.2011.01.111 21295551

[25] Vlassov AV, Magdaleno S, Setterquist R, Conrad R. Exosomes: current knowledge of their composition, biological functions, and diagnostic and therapeutic potentials. Biochimica et biophysica acta. 2012;1820(7):940–8. 10.1016/j.bbagen.2012.03.017 22503788

[26] Liu Y, Lai L, Chen Q, Song Y, Xu S, Ma F, et al MicroRNA-494 is required for the accumulation and functions of tumor-expanded myeloid-derived suppressor cells via targeting of PTEN. J Immunol. 2012;188(11):5500–10. 10.4049/jimmunol.1103505 22544933

[27] Fabbri M, Paone A, Calore F, Galli R, Gaudio E, Santhanam R, et al MicroRNAs bind to Toll-like receptors to induce prometastatic inflammatory response. Proc Natl Acad Sci U S A. 2012;109(31):E2110–6. 10.1073/pnas.1209414109 22753494 PMC3412003

